# Drivers of HIV self-testing among female sex workers: Findings from a multi-state study in Malaysia

**DOI:** 10.3389/fmed.2023.1022746

**Published:** 2023-04-06

**Authors:** Rayne S. Kim, Jeffrey A. Wickersham, Francesca Maviglia, Jonathan M. Galka, Iskandar Azwa, Kamal Gautam, Roman Shrestha

**Affiliations:** ^1^Section of Infectious Diseases, Department of Internal Medicine, Yale School of Medicine, New Haven, CT, United States; ^2^Department of the History of Science, Harvard University, Cambridge, MA, United States; ^3^Infectious Diseases Unit, Department of Medicine, Faculty of Medicine, University of Malaya, Kuala Lumpur, Malaysia; ^4^Department of Allied Health Sciences, University of Connecticut, Storrs, CT, United States

**Keywords:** HIV, self-testing, HIV prevention, female sex workers, key populations, Malaysia

## Abstract

**Background:**

Although research on HIV self-testing (HIVST) has rapidly increased, few studies have explored HIVST uptake in female sex workers (FSW), and none in Malaysia. Therefore, we endeavored to assess the willingness to use HIVST in this at-risk, vulnerable population.

**Methods:**

A cross-sectional survey study was conducted among 113 HIV-negative Malaysian FSW in 2017. Participants were recruited using advertisements on social media, flyers, and direct referrals from community-based organizations. Data were collected using self-administered surveys. Multivariable logistic regression was used to identify correlates of willingness to use HIVST.

**Results:**

Nearly a third of participants (30.1%) reported they would be willing to use HIVST. Multivariable analyses adjusting for Malay ethnicity, stable housing, living in Kuala Lumpur (KL), years in sex work, age of first sex work, childhood sexual assault, history of HIV testing, and previously in prison indicated that living in KL [adjusted odds ratio (aOR) = 5.214, *p* = 0.0137] was associated with a greater willingness to use HIVST. In contrast, having stable housing (aOR = 0.100, *p* = 0.0064) was negatively associated with willingness to use HIVST.

**Conclusions:**

Our results indicate that HIVST may potentially enhance the uptake of HIV testing among FSWs living in an urban environment and experiencing unstable housing, but an overall willingness to use HIVST is low. These findings highlight the need for efforts to increase awareness of HIVST among FSWs and additional research on the effective implementation of HIVST for FSW.

## 1. Introduction

With myriad factors such as dangerous working conditions, barriers to negotiating safe sexual practices, and unequal access to appropriate health services, female sex workers (FSW) are a key population at risk for HIV (1). Recent studies have indicated significantly higher levels of HIV among women engaged in sex work, with FSWs 13.5 times more likely to be infected with HIV than other women of reproductive age in low and middle-income countries (LMIC) (2). Additionally, sex workers (both cis and transgender women) are also at an increased risk of experiencing violence and homicide, other sexually transmitted infections (STI), and poorer mental health than their non-sex-working counterparts (3, 4). These harms frequently experienced by sex workers are caused and exacerbated by social marginalization and the criminalization of sex work (5).

In Malaysia, recent estimates indicate HIV prevalence among FSW is between 6.3% and 11.1% (6, 7). While this estimate is lower than HIV prevalence among other at-risk populations, such as MSM, it is more than ten times that of the general population (8). Sex work and the frequent co-occurrence of substance use are major HIV risk factors for women (7, 9), especially in Malaysia, which has become a major destination for sex tourism. Although there is a public health imperative to reduce HIV infections in at-risk populations [e.g., men who have sex with men (MSM), sex workers, people who use drugs], often, criminalization and discrimination drive such at-risk populations away from essential services. The criminalization of people at higher risk of infection, including FSW, discourages them from using HIV prevention and treatment services, increasing their vulnerability to HIV, as well as stigma, discrimination, marginalization, and gender-based violence (10). In recent years, acceptability for HIV preventative methods has been assessed in other at-risk groups in Malaysia, including MSM and transgender women (TW); however, there remains a dearth of literature assessing HIV prevention in FSW (11, 12).

HIV testing is paramount for HIV prevention and treatment options. Testing allows individuals to know their serological status and consequently be able to access HIV prevention options, including pre-exposure prophylaxis (PrEP), if HIV-negative, or be connected to HIV treatment options such as antiretroviral therapy (ART) promptly, if HIV-positive. Despite recommendations by international agencies such as the WHO and UNAIDS to implement innovative approaches to HIV prevention, uptake of HIV testing in Malaysia has been woefully inadequate, especially among women (1, 6). New approaches to addressing this disparity are needed. One such approach is HIV self-testing (HIVST), a process in which an individual performs an HIV rapid diagnostic test and views the result in private. Not only is HIVST highly accepted among a broad range of communities and cost-effective (13), it can be a source of empowerment among marginalized and high-risk groups with limited access to traditional HIV care services. As HIVST provides privacy and convenience, HIVST may be beneficial in FSW communities in the Malaysian context.

Research on HIVST implementation has rapidly increased; however, few studies have explored self-testing in FSW. The Malaysian Ministry of Health (MoH) has called for the advancement of the HIVST policy to improve the uptake of HIV testing among high-risk populations (6). Therefore, further information regarding HIVST uptake factors will be essential for implementing HIVST services for FSW in Malaysia. Although there is a recent study that explores HIVST in transgender women in Malaysia, there is considerable variation in uptake and factors that may impact uptake among individuals involved in sex work; it had been previously found that FSW were less likely to have ever been HIV tested than TW (7, 14). Therefore, we endeavored to assess the willingness to use HIVST in FSWs.

## 2. Methods

### 2.1. Study design and participants

The data used in this study came from a cross-sectional survey of FSW in Malaysia conducted in 2017 to explore perceptions and willingness to use different HIV prevention strategies, including PrEP and HIVST, and other general health-related measures. Participants were eligible to enroll if they were: (1) 18 years of age or older; (2) able to give informed consent; (3) assigned female at birth, identifying as a woman, and engaged in sex work; (4) living in Malaysia; and (5) able to read or speak Bahasa Malaysia, Tamil, or English.

### 2.2. Study procedures

Using convenience sampling, participants were recruited across Malaysia, including Selangor (inclusive of the Greater Kuala Lumpur region), Penang, and Seremban. Convenience sampling included posting advertisements in areas frequented by FSW, on social media, and direct referrals from staff members at community-based organizations serving the FSW community. Trained community-based research assistants screened potential participants in a private room and explained the study objectives and procedures to participants.

A total of 113 participants were enrolled in the study, and their participation was anonymous. Surveys were self-administered in Bahasa Malaysia and Tamil. Surveys lasted approximately 20 minutes, and data were collected using Qualtrics (Qualtrics, Provo, UT, USA) on a tablet or laptop computer. All study materials were translated from English to Bahasa Malaysia and Tamil, and written consent was obtained prior to completing the survey. Participants were compensated 20 RM (approximately 5 USD) for their time. The study was approved by the Institutional Review Boards at Yale University and the University of Malaya.

### 2.3. Measures

The primary outcome, willingness to use HIVST, was measured using a single-item question, “*Would you be willing to use HIV self-testing?*” with a binary response option of “yes” or “no.” Before answering this question, all participants read a brief description of HIVST, which explained what HIVST is and how it differs from traditional venue-based HIV testing.

Variables were selected based on a review of the relevant literature and knowledge of the Malaysian context. Participant characteristics included age, ethnicity, religion, relationship status, educational status, income, and living status.

Sexual risk behaviors included engagement in sex work, total years worked in sex work, age of initiation of sex work (“*How old were you when you first engaged in sex work?*”), number of sex work clients per day, use of mobile phones or mobile apps to solicit sex work clients (“*Have you ever used a mobile phone or mobile app to find clients for sex work?*”), use of street-based solicitation to recruit sex work clients, and engagement in condomless sex (CS) in the past 6 months (“*In the past 6 months, have you had anal or vaginal sex without using a condom?*”).

Drug and alcohol use measures include recent (past 30 days) use of alcohol, heroin, and the use of amphetamine-type stimulants (ATS). Prior injection of illicit drugs was also measured for the lifetime. Experience of childhood physical and sexual violence and adulthood physical violence was measured using questions from the US Centers for Disease Control and Prevention’s Behavioral Risk Factor Surveillance System (BRFSS) questionnaire for violence and victimization. Criminal justice history was measured using two variables, including previously in lock-up or jail and previously in prison (“*Have you ever been detained by the police, placed in lock-up, or imprisoned for any reason?*”).

General and sexual health measures were also included. Prior HIV testing was measured for the lifetime and the last 12 months. Prior STI testing (i.e., syphilis, chlamydia, and/or gonorrhea) was measured for a lifetime. A recent doctor visit was characterized as having been examined by a medical doctor for any reason in the last 12 months. Current depression was screened using the 10-item Clinical Epidemiological Scale-Depression (CES-D) (15), with scores of ≥10 on the CES-D indicating moderate to severe depression symptoms.

### 2.4. Analyses

Estimates were evaluated for statistical significance based on 95% Confidence Intervals (CI) and statistical significance of *p* < 0.05. We used descriptive statistics to summarize the characteristics of the sample. We then conducted bivariate logistic regression analyses between each independent variable and the primary outcome, willingness to use HIVST. Bivariate associations significant at *p* < 0.10 were selected for inclusion in the multivariate logistic regression analyses. Childhood physical violence and previously in lock-up were excluded from the final model due to their multicollinearity with childhood sexual assault and previously in prison, respectively. Analyses were performed using SAS version 9.4 (SAS Institute Inc., Cary, NC, USA).

## 3. Results

### 3.1. Participant characteristics

Table 1 shows participants’ characteristics stratified by the willingness to use HIVST. About half of the participants were Malay (55.8%), and over three-quarters had less than a high school education (75.2%). Almost half reported an income of ≥1,000 RM per month (47.8%), that they lived alone (49.6%), and more than half reported being single (63.7%). Most of the participants reported having stable housing (87.6%).

**TABLE 1:** Characteristics of participants stratified by their willingness to use HIV self-testing.

Variables	*N* (%) *N* = 113	Willingness to use HIVST		OR (95% CI)	*p*
		No (*n* = 79)	Yes (*n* = 34)		
Socio-demographics					
Age (years)	45.7 ± 11.3	45.1 ± 11.6	47.2 ± 10.8	1.017 (0.981–1.054)	0.3529
Ethnic origin: Malay	63 (55.8)	40 (35.4)	23 (20.4)	2.04 (0.877–2.737)	0.0977
Religion: Islam	69 (61.1)	46 (40.7)	23 (20.4)	1.50 (0.643–3.496)	0.3477
Education (form 5 level or higher)	28 (24.8)	20 (17.7)	8 (7.1)	0.908 (0.354–2.326)	0.8401
Income: = 1,000 RM MYR per month	54 (47.8)	36 (31.9)	18 (15.9)	1.344 (0.600–3.008)	0.4724
Relationship status: Single	72 (63.7)	50 (44.3)	22 (19.5)	1.063 (0.459–2.461)	0.8859
Living alone	56 (49.6)	43 (38.1)	13 (11.5)	0.705 (0.297–1.678)	0.4299
Stable housing	99 (87.6)	76 (67.3)	23 (30.4)	0.083 (0.021–0.321)	0.0003
Living in KL	69 (61.1)	40 (35.4)	29 (25.7)	0.598 (0.373–0.957)	0.0012
**Sexual risk behaviors**					
Years in sex work	*n* = 106				
<15	44 (41.5)	35 (33.0)	9 (8.5)	–	
Between 15 and 30	33 (31.1)	24 (22.6)	9 (8.5)		
30+	29 (27.3)	15 (14.1)	14 (13.2)	3.630 (1.292–10.197)	0.0144
Age of first sex work (years)	*n* = 106				
≤18	18 (17.0)	8 (7.5)	10 (9.4)	–	
18+	88 (83.0)	66 (62.3)	22 (20.7)	0.267 (0.094–0.760)	0.0134
Average # clients per day (last month)	4.3 ± 5.9	4.8 ± 6.6	3.1 ± 3.6	0.936 (0.850–1.030)	0.1753
Average # days engaged in sex work (last month)	11.7 ± 9.6	12.4 ± 9.5	10.1 ± 9.8	0.974 (0.932–1.017)	0.2340
Sex work 10+ days per month	64 (56.6)	48 (42.5)	16 (14.2)	0.574 (0.255–1292)	0.1797
Condomless sex	17 (15.0)	9 (8.0)	8 (7.0)	2.393 (0.835–6.863)	0.1022
Used street-based solicitation to recruit clients	15 (13.3)	9 (8.0)	6 (5.3)	0.609 (0.243–1.528)	0.2912
Used phone or internet to recruit clients	19 (16.8)	11 (9.7)	8 (7.1)	1.902 (0.688–5.258)	0.2150
**Violence and victimization**					
Childhood physical assault	31 (27.4)	17 (15.0)	14 (12.4)	2.553(1.071–6.085)	0.0344
Childhood sexual assault	32 (28.3)	17 (15.0)	15 (13.3)	2.879 (1.214–6.830)	0.0164
**Criminal justice history**					
Previously in lock-up	72 (63.7)	45 (39.8)	27 (23.9)	2.914 (1.135–7.483)	0.0262
Previously in prison	68 (60.2)	41 (36.3)	27 (23.9)	3.575 (1.395–9.162)	0.0080
**General and sexual health**					
HIV tested (ever)	95 (84.1)	63 (55.8)	32 (28.3)	4.061 (0.879–18.756)	0.0726
HIV tested (last 12 months)	75 (66.4)	53 (46.9)	22 (19.5)	0.899 (0.386–2.095)	0.9058
Other STI diagnoses (ever)	8 (7.1)	4 (3.5)	4 (3.5)	2.500 (0.587–10.647)	0.2153
Seen by a doctor in the last 12 months	99 (87.6)	70 (62.0)	29 (25.7)	0.746 (0.230–2.417)	0.6247
Current depressive symptoms	79 (69.9)	51 (45.1)	28 (24.8)	2.562 (0.947–6.929)	0.0638
**Alcohol/drug use (last 30 days)**					
Alcohol	12 (10.6)	9 (8.0)	3 (2.7)	0.753 (0.191–2.972)	0.6852
Heroin	9 (8.0)	7 (6.2)	2 (1.8)	0.634 (0.125–3.223)	0.5830
Crystal meth	25 (22.1)	16 (14.2)	9 (8.0)	1.395 (0.545–3.569)	0.4873
Injection drug use	10 (8.9)	3 (2.7)	7 (6.2)	2.000 (0.352–11.363)	0.4343

Regarding sexual risk behaviors, 58.4% of participants reported having worked in sex work for more than 15 years and reported an average of 4.3 clients per day during the last month. More than half (56.5%) reported working 10+ days per month in sex work. Most reported having been tested at least once in their lifetime (84.1%), with 66.4% having been HIV tested in the last 12 months. Regarding violence and victimization, nearly one-third reported having experienced physical assault (27.4%) and sexual assault (28.3%) during childhood. Additionally, more than half reported being previously locked up (63.7%) and imprisoned (60.2%).

### 3.2. Willingness to use HIVST

Nearly a third of participants (30.1%) were willing to use HIVST. Table 1 shows the bivariate correlates of willingness to use HIVST. Years of sex work for over 30 years (OR = 3.630, *p* = 0.014), experience of childhood physical (OR = 2.914, *p* = 0.026) and sexual (OR = 2.878, *p* = 0.016) assault, and previously being in lock-up (OR = 2.914, *p* = 0.026) and prison (OR = 3.575, *p* = 0.008) were significantly positively associated with willingness to use HIVST, while stable housing [odds ratio (OR) = 0.083, *p* < 0.001], living in Kuala Lumpur (OR = 0.598, *p* = 0.001) and age of first sex work ≤18 years (OR = 0.267, *p* = 0.013) were significantly negatively associated with willingness to use HIVST in bivariate analyses at *p* < 0.05. Malay ethnicity (OR = 2.04, *p* = 0.098) and history of lifetime HIV testing (OR = 4.061, *p* = 0.073) were significantly positively associated with willingness to use HIVST at *p* < 0.10.

Table 2 shows the independent correlates associated with willingness to use HIVST in multivariable regression. Living in KL [adjusted OR (aOR) = 4.486, *p* = 0.019] was associated with a greater willingness to use HIVST, whereas stable housing (aOR = 0.101, *p* = 0.008) was associated with a lower willingness to use HIVST.

**TABLE 2:** Multivariate logistic regression analysis of factors associated with willingness to use HIV self-testing (*N* = 113).

	Willingness to use HIVST		
Variables	aOR	95% CI	*p*
Malay	1.828	0.615-5.435	0.2776
Stable housing	0.101	0.019-0.542	0.0075
Living in KL	4.486	1.294-18.148	0.0191
Years in sex work: 30+	2.201	0.647-7.492	0.2068
Age of first sex work: ≤18 years	2.922	0.808-10.567	0.1020
Childhood sexual assault	1.325	0.406-4.324	0.6405
Previously in prison	1.611	0.437-5.946	0.4738
HIV tested (ever)	2.809	0.360-21.885	0.3242
Current depressive symptoms	1.049	0.285-3.857	0.9425

## 4. Discussion

To our knowledge, this is the first study to assess willingness to use HIVST among FSW in Malaysia, a key population with high vulnerability to HIV. The subset of FSW analyzed in this study was taken from the same sample of a previous published article studying transgender women (14). The complete sample collected from this survey includes both transgender and cisgender FSW, and these analyses focus specifically on cisgender FSW. Our findings indicate that FSW displayed a low willingness to use HIVST (30.1%), particularly when compared with findings from other settings and populations (14, 16, 17). Notably, this sample of cisgender FSW had a lower willingness to use HIVST than TW in the same sample (47.6%) (14). The relatively low level of willingness observed in this study may be due to participants being recruited from venues where traditional HIV testing services are routinely provided to FSW. Although global evidence indicates that HIV testing uptake is low among FSW due to a mix of structural barriers to access and stigma surrounding both HIV and sex work (18), our findings indicate that over most (84.5%) of our sample had received an HIV test in their lifetime, with more than half of participants having been tested recently. Thus, FSW in this sample may already be comfortable with their current modality of HIV testing. The difference of willingness to use HIVST between cisgender FSW and transgender FSW suggest that cisgender FSW may feel more comfortable than TW (14). Additionally, although not assessed in the current study, various issues related to lack of awareness about the test, associated cost, administration of the test, accuracy of the test, pre- and post-counseling, potential mental health outcomes of those with a preliminary positive HIVST result, and access to confirmatory testing and referrals may explain the low willingness to use HIVST in this sample (19, 20).

Overall, our sample exhibited high levels of access to health care (87.6% had been seen by a doctor in the last year) and low levels of condomless sex (15.0% in the last 6 months) compared to previous studies with Malaysian FWS (7, 21); this is likely explained by choice of the recruitment venue. However, our sample reported high levels of psychosocial needs: a majority (69.9%) were experiencing depressive symptoms at the time of the survey, and a significant number of participants reported a history of physical (27.4%) and sexual (28.3%) assault. These findings are like that for the TW as well (14). Furthermore, most participants reported having been previously detained (63.7%) or incarcerated (60.2%). Previous studies have supported that incarceration history, regardless of gender, is associated with increased HIV-risk-related behaviors (22, 23). In Malaysia, our findings suggest that FSW experience risk factors such as incarceration and injection drug use, all at much higher rates than the data in recent studies assessing other at-risk populations such as MSM and TW (11, 12, 14). About half of the participants (47.8%) were living with less than 1,000 RM MYR per month, less than half of the median monthly income in Malaysia, which at the time the study was conducted was equal to 2,160 RM MYR (24). These findings confirm that the criminalization and stigma surrounding sex work result in high levels of socio-economic marginalization of sex workers; while access to services through community-based organizations serving the FSW community appears to mitigate barriers in access to healthcare and engagement in condomless sex, a high level of psycho-social needs remain unaddressed.

We found that willingness to use HIVST was positively associated with FSW participants reporting living in Kuala Lumpur. Although this finding contrasts with a prior study done with TW in Malaysia (14), there is a large body of evidence that suggests HIV testing frequencies are significantly higher among people who live in urban areas compared to those who live in rural areas (25, 26), due to lower availability of services and greater transportation barriers to health care access in rural areas (27). It is possible that FSWs from urban areas may perceive HIVST to offer them with more privacy and control over HIV testing experience. Similarly, they may have perceived HIVST as a more convenient and time-efficient option compared to traditional HIV testing services, thus making HIVST more appealing to them. While HIVST could help overcome some of the access barriers faced by rural individuals, our findings show low willingness to use HIVST among rural FSW: this paradox represents a challenge to the use of HIVST as an intervention to increase uptake among rural populations and indicates that greater efforts to increase HIV prevention awareness and uptake among rural FSW are needed. Nonetheless, our findings are encouraging as far as HIV prevention among urban FSW is concerned since existing literature from other settings indicates that urban populations exhibit higher HIV prevalence than rural populations (28). Given that individuals in urban settings are at increased risk, the higher willingness to use HIVST provides an additional prevention strategy in promoting public health practice among urban FSWs.

Additionally, we found that the willingness to use HIVST was negatively associated with FSW participants who reported stable housing. Research indicates that individuals with stable housing were less likely to engage in sexual risk behaviors and substance abuse (23); thus, this result could be due to lower self-perceived HIV risk by FSW with stable housing. Moreover, unstable housing conditions have been shown to represent a structural barrier to HIV risk reduction by hindering FSW’s agency over their sexual and HIV-related behavior (29, 30). Therefore, the higher willingness to use HIVST among unstably housed FSW is very promising for HIV prevention efforts in this subpopulation.

In interpreting these findings, certain limitations of our study should be considered. This study relied on cross-sectional data, limiting our ability to infer causal directions underlying the observed associations. Many participants were recruited from community-based organizations receiving HIV testing services, which may account for the higher rate of recent venue-based HIV testing and lower interest in HIVST. Many participants were also recruited from brothels, and the consequences associated with legal and policing risks of this occupational environment have been found to increase treatment-seeking behavior (31). Moreover, our participants were recruited using direct referrals from staff members of the local community-based organizations. This convenience sampling and relatively small sample size may have limited the statistical power of our analyses and may have produced a sample population not representative of the broader population of FSW in Malaysia. Although this study may be more representative of care-seeking populations, this study is the largest and most geographically distributed sample of FSW published in Malaysia. Furthermore, the findings reported in this study are constrained by the lack of contextual information about participants’ previous knowledge of HIVST, previous use of HIVST, or other aspects of HIVST (e.g., the preferred method of distribution, cost, type of kit, and pre-and post-test counseling and linkage to prevention/treatment services). These factors are likely important predictors of willingness and should be examined by future research to inform efforts to scale up interventions utilizing HIVST. Additionally, as all data were collected through self-report, there is potential for social desirability bias and recall bias.

## 5. Conclusion

The findings from this study provide essential data regarding FSW’s willingness to use HIVST in Malaysia. The findings in this study offer a mild contrast to the small but growing body of evidence that suggests HIVST has the potential to enhance uptake and retention of HIV testing in FSWs. HIVST, as a very convenient and private method of HIV prevention, may be most beneficial for FSW who are concerned about their privacy, safety, discrimination, and criminalization; however, our findings suggest that most Malaysian FSWs may not be willing to use HIVST. More research is needed to explore this due to lack of education on HIVST, preference for in-person testing and counseling, or other barriers. As the MoH is developing guidelines for HIV self-testing, the findings from this study could begin to inform program planning, scale-up, and implementation, but our results also suggest further engagement with communities of FSWs to understand their preferences and priorities is crucial. Advances in HIV prevention science present a promising future in HIV prevention efforts. HIVST serves as a promising addition to interventions that could make HIV services more targeted to the needs of key populations at risk for HIV, including FSW; however, what remains missing is a respectful and inclusive response to marginalized and vulnerable populations, including sex workers.

## Data availability statement

The raw data supporting the conclusions of this article will be made available by the authors, without undue reservation.

## Ethics statement

This study was approved by the Institutional Review Boards at Yale University and the University of Malaya. The patients/participants provided their written informed consent to participate in this study.

## Author contributions

RK, JW, and RS contributed to the conception and design of the study. JW and JG organized the database. RK, FM, and KG performed the statistical analysis. RK wrote the first draft of the manuscript. All authors contributed to manuscript revision, read, and approved the submitted version.
